# X-Linked Lymphoproliferative Disease Mimicking Multisystem Inflammatory Syndrome in Children—A Case Report

**DOI:** 10.3389/fped.2021.691024

**Published:** 2021-08-03

**Authors:** Seraina Prader, Nicole Ritz, Frédéric Baleydier, Maya C. Andre, Noémie Stähli, Kevin Schmid, Hanna Schmid, Andreas Woerner, Tamara Diesch, Patrick M. Meyer Sauteur, Johannes Trück, Fabienne Gebistorf, Lennart Opitz, Michael P. Killian, Tommaso Marchetti, Stefano Vavassori, Géraldine Blanchard-Rohner, Valerie Mc Lin, Serge Grazioli, Jana Pachlopnik Schmid

**Affiliations:** ^1^Division of Immunology, University Children's Hospital Zurich, Zurich, Switzerland; ^2^Pediatric Infectious Diseases and Vaccinology, University of Basel Children's Hospital Basel, Basel, Switzerland; ^3^Department of Pediatrics, The Royal Children's Hospital Melbourne, The University of Melbourne, Melbourne, VIC, Australia; ^4^Pediatric Hemato-Oncology Unit, Department for Women, Children, and Adolescents, University Hospitals Geneva, Geneva, Switzerland; ^5^CANSEARCH Research Laboratory, Medical Faculty, Geneva University, Geneva, Switzerland; ^6^University Children's Hospital, Division of Respiratory and Critical Care Medicine, University of Basel, Basel, Switzerland; ^7^Emergency Department, University Children's Hospital Zurich, Zurich, Switzerland; ^8^Department of Pediatric and Neonatal Intensive Care, University Children's Hospital Zurich, Zurich, Switzerland; ^9^Division of Pediatric Rheumatology, University of Basel Children's Hospital Basel, Basel, Switzerland; ^10^Division of Pediatric Hematology/Oncology, University Children's Hospital of Basel, Basel, Switzerland; ^11^Division of Infectious Diseases and Hospital Epidemiology, University Children's Hospital Zurich, Zurich, Switzerland; ^12^Division of Neonatal and Pediatric Intensive Care, University Hospitals of Geneva, Geneva, Switzerland; ^13^Functional Genomic Center Zurich, University of Zurich and Swiss Federal Institute of Technology in Zurich, Zurich, Switzerland; ^14^Unit of Immunology and Vaccinology, Division of General Pediatrics, Department of Woman, Child, and Adolescent Medicine, Geneva University Hospitals and Faculty of Medicine, University of Geneva, Geneva, Switzerland; ^15^Swiss Pediatric Liver Center, Department for Women, Children, and Adolescents, University Hospitals Geneva, Geneva, Switzerland; ^16^Medical Faculty, University of Geneva, Geneva, Switzerland; ^17^University of Zurich, Zurich, Switzerland

**Keywords:** case report, fatal, pediatric, XLP1, HLH, SARS-CoV-2, MIS-C, PIMS-TS

## Abstract

Most children with a SARS-CoV-2 infection are asymptomatic or exhibit mild symptoms. However, a small number of children develop features of substantial inflammation temporarily related to the COVID-19 also called *multisystem inflammatory syndrome in children* (MIS-C) or *pediatric inflammatory multisystem syndrome temporally associated with SARS-CoV-2* (PIMS-TS), clinically similar to Kawasaki disease, toxic shock syndrome and hemophagocytic lymphohistiocytosis (HLH). It is well-known that genetic pre-disposition plays an important role in virally-triggered diseases such as Epstein-Barr virus (EBV)-associated HLH, while this has not yet been established for patients with MIS-C. Here we describe a male patient fulfilling the diagnostic criteria of MIS-C, who was initially treated according to current consensus guidelines. Presence of hypofibrinogenemia, normal lymphocyte counts and C-reactive protein, but substantial hyperferritinemia distinguish this patient from others with MIS-C. The clinical course following initial presentation with acute respiratory distress syndrome was marked by fatal liver failure in the context of EBV-associated HLH despite treatment with steroids, intravenous immunoglobulins, interleukin (IL)-1 receptor blockade and eventually HLH-directed treatment. X-linked lymphoproliferative disease type 1 (XLP1), a subtype of primary HLH was diagnosed in this patient post-mortem. This case report highlights the importance of including HLH in the differential diagnosis in MIS-C with severe disease course to allow specific, risk-adapted treatment and genetic counseling.

## Clinical Implications

Laboratory parameters such as level of fibrinogen, ferritin, C-reactive protein and lymphocyte counts may help to distinguish Hemophagocytic lymphohistiocytosis (HLH) from COVID-19-related multisystem inflammatory syndrome in children (MIS-C).

## Background

Most children with a SARS-CoV-2 infection are asymptomatic or exhibit mild symptoms (1, 2). However, in April 2020, a rare but serious clinical entity has been described in children, encompassing symptoms of hyperinflammation, resembling toxic shock syndrome (TSS) or Kawasaki disease (KD) (3–5), now generally referred to as either *multisystem inflammatory syndrome in children* (MIS-C) or *pediatric inflammatory multisystem syndrome—temporally associated with SARS-CoV-2* (PIMS-TS). In May 2020, the World Health Organization (WHO) has published a MIS-C case definition for patients under the age of 19 years with fever ≥3 days and fulfilling 4 out of the 4 following criteria: (i) at least two clinical signs of multisystem inflammatory involvement (ii) elevated markers of inflammation (iii) no other microbial cause of inflammation—including bacterial sepsis and TSS—and (iv) evidence of current or past SARS-CoV-2 infection (https://www.who.int/publications/i/item/multisystem-inflammatory-syndrome-in-children-and-adolescents-with-covid-19). Some of the typical laboratory findings of MIS-C include elevated C-reactive protein (CRP), ferritin, D-dimers and fibrinogen, troponin and N-terminal pro brain natriuretic peptide (NT-pro BNP), high neutrophil to lymphocyte ratio and low platelets (6).

Hemophagocytic lymphohistiocytosis (HLH) resembles MIS-C. Diagnosis of HLH is defined by the presence of 5 out of the following 8 criteria: (i) fever, (ii) hemophagocytosis in bone marrow or other organs, (iii) bicytopenia, (iv) enlarged spleen, (v) ferritin > 500 ug/L, (vi) soluble IL-2 receptor >2,400 U/mL, (vii) hypofibrinogenemia or hypertriglyceridemia, and (viii) decreased NK-cell cytotoxicity (7). HLH may be secondary to other diseases, but in every patient fulfilling the diagnostic criteria of HLH, inborn defects in lymphocyte cytotoxic activity should be considered, also known as primary HLH (8). This is paramount given that early diagnosis is crucial for a successful outcome. Primary HLH includes X-linked lymphoproliferative disease (XLP1)—a rare immunodeficiency characterized by immune dysregulation—caused by mutations in the SH2 domain–containing protein 1A *(SH2D1A)* gene, which encodes signaling lymphocytic activation molecule (SLAM)–associated protein (SAP). Clinical manifestations of XLP1 include lymphoma, dysgammaglobulinemia and HLH, the latter often triggered by Epstein-Barr virus (EBV) infection (9).

## Clinical Communication

Here we report a case of fatal HLH with liver failure, at first considered to be MIS-C, in a patient tested positive for COVID-19, later diagnosed with EBV infection and underlying XLP1.

**Figure 1:**
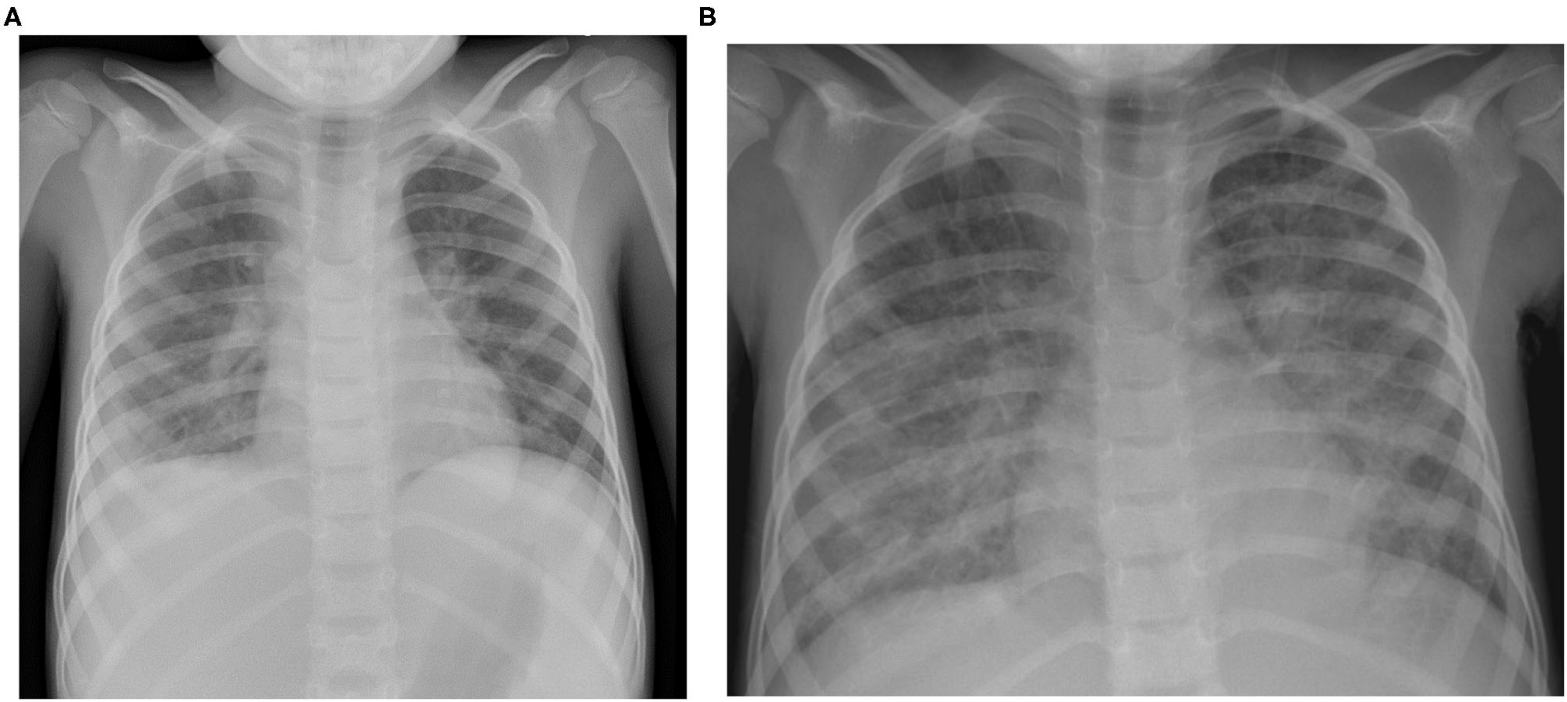
Chest X ray at the day of admission **(A)** showing diffuse bilateral interstitial markings and obscured contour of the right diaphragm border as a sign of consolidation/pleural effusion and one day after admission **(B)** showing rapidly evolving bilateral alveolar opacities compatible with pulmonary edema next to the known right-sided lobar infiltrate.

The previously healthy 6-year-old boy presented at our emergency department after a history of 10 days of fever. Five days earlier, the diagnosis of a SARS-CoV-2 infection had been made through a positive reverse transcription polymerase chain reaction (RT-PCR) from a nasopharyngeal swab. On admission, physical exam was remarkable for fever (39.8°C), tachycardia (150 bpm), hypotension (75/38 mmHg), tachypnea (50/min), and abdominal pain (Table 1). Chest radiography was compatible with right lower lobe pneumonia and moderate pleural effusion (Figure 1A). Abdominal ultrasound revealed hepatosplenomegaly with ascites. Echocardiographic investigation showed normal cardiac function and absence of coronary abnormalities. Blood tests revealed anemia (hemoglobin 108 g/L), thrombocytopenia (103 G/L), and normal neutrophil and lymphocyte counts (Table 2). While CRP was normal, ESR (36 mm/h) and ferritin (3,995 ug/L) were elevated. Liver parameters were abnormal (ALT 511 U/L, bilirubin 75 umol/L, GGT (gamma-glutamyltransferase) 396 U/L, and albumin 22 g/L) and there was profound hyponatremia (124 mmol/L). Troponin and NT-pro BNP were normal. Fibrinogen was decreased (1.09 g/L) with elevated D-dimers (6.4 mg/L). Multiplex PCR was negative for respiratory viruses including SARS-CoV-2 on admission and SARS-CoV-2 antibodies were later found to be absent.

**Table 1:** Clinical characteristics: patient with XLP1 compared to MIS-C.

	**Patient**	All MIS-C cases, *n* = 58, %, median *Whittaker et al. (6)
Age in years at diagnosis	6	9
**Sex**		
Male	X	43
Female		57
**Race**		
Black		38
Asian		31
White	X	21
Other		10
**Clinical features at presentation**		
Abdominal pain	X	53
Diarrhea	.	52
Rash	.	52
Shock	X	50
Vomiting	.	45
Conjunctival injections	.	45
Mucous membrane changes	.	29
Headache	.	26
Respiratory symptoms	X	21
Lymphadenopathy (incl. hepatosplenomegaly)	X	16
Swollen hands and feet	.	16
Sore throat	.	10
Confusion	.	9
**Cardiac/circulatory/kidney**		
Acute kidney injury	Normal creatinine but proteinuria	22
Inotropic support	.	47
ECMO	.	5
**Respiratory**		
Intubation	x (d + 7 after admission)	43
**Pharmacotherapy**		
IVIG	X	71
Corticosteroids	X	64
Anakinra	X	5
Infliximab	.	14
Antilymphocyte immunoglobulin	X	0
Ciclosporin	X	0
Rituximab	X	0
**No. of immunomodulatory treatments**		
2		60
3		16
>3	X	0
**Outcomes**		
Coronary artery aneurysm	.	14
Death	X (d + 8 after admission)	2

*ECMO, Extracorporeal membrane oxygenation; IVIG, intravenous immunoglobulins*.

**Table 2:** Laboratory results: patient with XLP1 compared to MIS-C.

	**Patient (on admission, first value resp.)**	**All MIS-C cases, median (IQR) (*n* = 58), % Whittaker et al. (6)**
**Virology**		
SARS-CoV-2 PCR positive	X (5 days prior positive testing)	26
SARS-CoV-2 IgM or IgG AB positive	.	83
Any SARS-CoV-2 PCR or IgM/IgG positive	X	78
No positive results	.	22
EBV PCR	20 × 10^6^ copies/mL	NA
**EBV IgM Positive**	X	NA
**Laboratory values**		
**Hematology**		Median value (IQR)
Leukocytes G/L	8.4	17 (12–22)
Neutrophils G/L	2.5	13 (10–19)
Lymphocytes G/L	4.3	0.8 (0.5–1.5)
Hemoglobin g/L	108	92 (83–103)
Platelets G/L	103	151 (104–210)
**Inflammatory markers**		
CRP mg/L	6.9	176 (156–338)
Ferritin ug/L	3,995	379 (359–1,280)
sIL-2 R ng/L	4,453	
ESR mm/h	36	NA
**Biochemistry**		
LDH U/L	876	419 (319–887)
ALT U/L	511	42 (26–95)
Bilirubin umol/L	75	NA
Lactate mmol/L	1.6	NA
Albumin g/L	22	24 (21–27)
Creatinine umol/L	36	71 (43–108)
Sodium mmol/L	124	NA
**Cardiac markers**		
Troponin ng/L	7	45 (8–294)
NT-pro-BNP ng/L	NA	788 (174–10548)
CK-MB ug/L	0.7	NA
**Coagulation**		
Fibrinogen g/L	1.09	5.7 (4.4–7)
D-Dimer mg/L	6.4	3.6 (2.1–8.2)
**Immunologic features d** **+** **7 after admission**		
**Lymphocyte flowcytometry**		
Lymphocytes G/L	8.49	NA
CD3+ G/L	5.69	NA
CD4+ G/L	4.09	NA
CD4+HLA-DR+ %	1	NA
CD8+HLA-DR+ %	5	NA
CD19+ G/L	1.02	NA
CD16+CD56+ G/L	1.1	NA
Perforin in CD8+ % (Ref > 5)	48	NA
Perforin in CD56+ % (Ref >5)	41	NA
NK cell degranulation CD107a % (Ref > 10)	40.5	NA

*Values marked in yellow differ between XLP1 patient and MIS-C and may help in distinguishing the diagnosis. EBV, Epstein Barr virus; SARS-CoV-2, severe acute respiratory syndrome coronavirus 2. CRP, common reactive protein; ESR, erythrocyte sedimentation rate; LDH, lactate dehydrogenase; ALT, alanine transaminase*.

The patient fulfilled the diagnostic criteria of MIS-C at time of admission and was transferred to intensive care. Empiric antibiotic treatment was started and, as he developed acute respiratory distress in the context of pulmonary edema (Figure 1B) and assumed MIS-C, treatment with intravenous immunoglobulins (IVIG; 2 g/kg) and methylprednisolone (30 mg/kg/day) was initiated. Twenty-four hours later, in the absence of clinical improvement, therapy was escalated with interleukin (IL)-1 receptor antagonist anakinra (8–9 mg/kg/day s.c.). The patient's clinical condition continued to worsen. He developed liver failure, lactacidemia and profound coagulopathy, highly suggestive of HLH. Therefore, anakinra was stopped and treatment with anti-human-T-lymphocyte-immunoglobulin (ATG) and ciclosporin initiated. Suspicion of primary HLH was something later strengthened by the detection of very high EBV copy numbers in the blood (20 × 10^6^ copies/mL). Rituximab was added once EBV results were available. Despite added support using molecular adsorbent recirculation system (MARS), the patient's condition further deteriorated and he succumbed on day 8 after admission. Immunological investigations on day 6 had shown normal CD107a expression in NK cell degranulation assay and normal intracellular perforin expression by flow cytometry. However, an X-linked mutation (c.163C > T; p.R55X; position on chrX:124365786; reference genome: GRCh38.p12) in the SAP*/* SH2 domain–containing protein 1A *(SH2D1A)* gene was found, which has previously been described to be disease-causing. Thus, the diagnosis of an X-linked lymphoproliferative syndrome type 1 (XLP1) was made post-mortem. The mother is carrier of a de novo heterozygous mutation. A detailed timetable of the patient's clinical history is shown in Table 3.

**Table 3:** Timeline patients history.

**d+**	**Clinical feature**	**Interventions**	**Laboratory features**	**Infectious diagnostics**	**Diagnostics**	**Immunosuppressive treatment**	**Antibiotics**	**Further treatment**
day 1-5	Fever							
6	Fever		Lymphocytopenia, CRP 12 mg/l	SARS CoV 2 RT-PCR positive nasopharyngeal swab				
7	Fever							
8	Fever							
9	Fever							
10	Visit GP because of fever, abdominal pain, tachypnoe transfer to hospital		Anemia, thrombocytopenia, normal CRP, elevated lactat dehydrogenase, liver enzymes, hypofibrinogenemia	Multiplex PCR for respiratory viruses negative SARS-CoV2 antibodies negative, blood cultures negative	Chest X ray - pleural effusion echocardiography normal cardiac function abdominal unltrasound: hepatosplenomegaly, ascites		Amoxicillin i.v.	
11	Fever, stable general condition, fever 38°	O2 supplementation 2 l/min	Hyponatremia		Chest X ray - signs of pulmonary edema, echocardiography normal cardiac function		Amoxicillin i.v.	
12	Uncontrolled fever (40°), increasingly unstable condition (O2 6 l/min)	O2 supplementation 6 l/min	Increasing liver enzymes, hypofibrinogenemia, hypalbuminemia, distured coagulation parameters			Methylprednisolone 30 mg/kg i.v.	Amoxicillin i.v.	IVIG 1 g/kgalbumin 20%furosemid i.v.
13	Uncontrolled fever, unstable condition	O2 supplementation 2 l/min	Ferritin 21'333 mcg/l		Echocardiography normal cardiac function	Methylprednisolone 30 mg/kg i.v., anakinra 4 mg/kg 2x/day s.c.	Amoxicillin i.v.	IVIG 1 g/kgalbumin 20%furosemid i.v.
14	Uncontrolled fever, unstable condition	O2 supplementation 2 l/min			Abdominal ultrasound with hepatosplenomegaly, cholecystitis	Methylprednisolone 30 mg/kg i.v. anakinra 3 mg/kg 3x/day s.c.	Amoxicillin i.v. stop pipercillin/tazobactam i.v.	IVIG 2 g/kgfurosemid i.v.
15	Acute and massive clinical detoriation		Elevated amoniak, lactatacedemia, decreased FV comparable with acute liver failure, creatinin elevation	EBV PCR 18584 x 10^8^ Geq/ml (blood)CMV PCR (reactivation) positive (blood and CSF)HHV6 (reactivation?) positive (CSF)		Methylprednisolone 10 mg/kg i.v. Anakinra 3 mg/kg 3x/day s.c. Rituximab i.v. Ciclosporine i.v.	Pipercillin/tazobactam i.v. gangciclovir, caspofungin	Erythrocyte/ thrombocyte transfusions
16		Intubation MARS continuous veno-venous hemodiafiltration (CVVHDF) plasmapheresis			Bone marrow punction: signs of macrophage activation and hemophagocytosis	Methylprednisolone 10 mg/kg i.v. Ciclosporine i.v., Grafalon i.v.	Pipercillin/tazobactam i.v. gangciclovir, caspofungin	Erythrocyte/ thrombocyte transfusions
17	Clinical signs of ammonium encephaloathy	MARScontinuous veno-venous hemodiafiltration (CVVHDF)plasmapheresis			Cranial CT: no edema	Methylprednisolone 10 mg/kg i.v. Ciclosporine i.v., Grafalon i.v.	Pipercillin/tazobactam i.v. gangciclovir caspofunginvancomycin	Erythrocyte/ thrombocyte transfusions
18	Bilateral mydriasis - redirection of care and exitus letalis							

## Discussion

Our patient initially presented with fever, pneumonia and rapidly evolving ARDS. Due to previous infection with SARS-CoV-2 the suspected initial diagnosis was MIS-C. He ultimately succumbed secondary to liver failure as a consequence of EBV-triggered HLH. These two disease entities, MIS-C and HLH, have an overlapping clinical spectrum but when comparing our patient's laboratory findings with the ones of a total of 58 previously described MIS-C patients (6), there were some key distinguishing findings: (i) Fibrinogen is usually elevated in MIS-C patients. This is in contrast to hypofibrinogenaemia found in our patient. (ii) MIS-C patients usually have low lymphocytes, while total lymphocyte counts were normal in our patient. (iii) Elevated levels of ferritin are common to both entities but, excessively high concentrations, with a 10-fold increase compared to MIS-C patients, were observed in our patient (6). (iv) All MIS-C patients reported by Whittaker et al. had CRP levels > 100 mg/L whereas our patient's CRP was normal, possibly secondary to liver failure.

It is important to evaluate clinical and laboratory findings in a patient with suspected MIS-C very carefully, as PCR might be already negative and you may have to wait for serological results.

Whether SARS-CoV-2 infection may have triggered this fulminant EBV infection is speculative: viral clearance could have occurred during these days. Serology might have remained negative, as dysgammaglobulinemia is a known feature in about one third of XLP1-patients.

Initial treatment for both diseases consists of corticosteroids, in MIS-C patients also of high-dose IVIG. Prior HLH treatment protocols have included IVIG too, so initial anti-inflammatory and immunomodulatory treatment regimens are similar. In patients with MIS-C not responding to initial treatment, escalation includes the use of biologicals, such as IL-1 or IL-6 blocking agents. For primary HLH, there are several treatment schemes available, including VP-16, alemtuzumab, anti- thymoglobulin and ciclosporin. If there is evidence of EBV-driven disease, treatment with a monoclonal anti-CD20 antibody (rituximab) has been used successfully. Recently, treatment with Janus kinase inhibitors (e.g., ruxolitinib) has also shown promising results (10). In primary HLH, the aim of treatment is to induce remission from the hyperinflammatory state, but only hematopoietic stem cell transplantation is curative.

It remains unclear whether our patient's SARS-CoV-2 infection had an impact on the course of disease. We believe that it was a simple coincidence, but it may be possible that the negative serology for SARS-CoV-2 (despite positive anti-EBV IgM) may be partly explained by the underlying disease, as antibody deficiency is a possible feature in XLP1 (9).

XLP1 may manifest in male patients at the same age as typical MIS-C and shares some clinical and laboratory features, especially at presentation. Clinical course and some laboratory parameters such as level of fibrinogen, ferritin, C-reactive protein and lymphocyte counts may help to distinguish HLH from MIS-C. Elevated aminotransferases in patients with MIS-C have been shown to be associated with more severe disease and higher inflammatory parameters, but acute liver failure in both, MIS-C and HLH, is very rare. Primary HLH and XLP1 in particular, should be part of the differential diagnosis of MIS-C. This can be rapidly evaluated by measuring SAP-expression by flow cytometry. It is absolutely necessary to distinguish MIS-C from primary HLH as prognosis and management differ. Curative treatment by hematopoietic stem cell transplantation should be considered in primary HLH and genetic counseling of affected families is needed.

## Methods

The parents have given consent and the patient was enrolled in an ongoing, institutional review board-approved study (PB_2016_02280) at the Division of Immunology at the University Children's Hospital Zurich, Switzerland; ClinicalTrials.gov. NCT02735824.

Whole exome sequencing and analysis of sequences have been performed as described previously (11).

## Data Availability Statement

The original contributions presented in the study are included in the article/supplementary materials, further inquiries can be directed to the corresponding author/s.

## Ethics Statement

Written informed consent was obtained from the minor(s)' legal guardian/next of kin for the publication of any potentially identifiable images or data included in this article.

## Author Contributions

SP wrote the manuscript. NR, FB, FG, GB-R, MA, AW, PM, VM, and SG contributed to patients' care, data collection, and interpretation. MK contributed to writing of the manuscript. SV and TM carried out functional analysis of lymphocytes. LO performed bioinformatics analyses of exome sequencing data. JP coordinated the study, was involved in the care of the family, and wrote the manuscript. All authors provided critical feedback and contributed to the final version of the manuscript.

## Conflict of Interest

JP is member of a data monitoring committee for Leniolisib for Novartis and advisory board for Emapalumab for SOBI. The remaining authors declare that the research was conducted in the absence of any commercial or financial relationships that could be construed as a potential conflict of interest.

## Publisher's Note

All claims expressed in this article are solely those of the authors and do not necessarily represent those of their affiliated organizations, or those of the publisher, the editors and the reviewers. Any product that may be evaluated in this article, or claim that may be made by its manufacturer, is not guaranteed or endorsed by the publisher.
